# Auditory and Olfactory Deficits in Essential Tremor – Review of the Current Evidence

**DOI:** 10.5334/tohm.57

**Published:** 2020-06-09

**Authors:** Yildizhan Sengul

**Affiliations:** 1 Department of Neurology, Gaziosmanpasa Training and Research Hospital, University of Medical Sciences, Gaziosmanpasa, Istanbul, TR

**Keywords:** essential tremor, non-motor features, sensory deficit, hearing impairment, olfactory dysfunction, neurodegenerative disease

## Abstract

**Background:**

Essential tremor (ET) is the most common adult movement disorder, characterized by several motor and increasingly well recognized non-motor symptoms. Sensory deficits, such as hearing impairment and olfactory dysfunction, are amongst them. This review analyzes the available evidence of these sensory deficits and their possible mechanistic basis in patients with ET.

**Method:**

A PubMed literature search on the topic was performed in the May 2019 database.

**Results:**

Nineteen articles on hearing impairment and olfactory dysfunction in ET patients were identified. The prevalence of hearing impairment is higher in ET patients than healthy controls or Parkinson disease. Cochlear pathologies are suggested as the underlying cause, but there is still a lack of information about retrocochlear pathologies and central auditory processing. Reports on olfactory dysfunction have conflicting results. The presence of mild olfactory dysfunction in ET was suggested. Conflicting results may be due to the lack of consideration of the disease’s heterogeneity, but according to recent data, most studies do not find prominent evidence of olfactory loss in ET.

**Conclusion:**

Although there is increasing interest in studies on non-motor symptoms in ET, there are few studies on sensory deficits, which are of particularly high prevalence. More studies are needed on to investigate the basis of non-motor symptoms, including sensory deficits.

## Introduction

Essential tremor (ET) is one of the most common movement disorders and the most common cause of tremor in adult life. The prevalence of ET has been estimated to be 4.0% – 5.6% among individuals age ≥ 40 years but continues to increase with age [1]. ET has previously been called “benign tremor” but the adjective “benign” has been removed due to the newly recognized neurodegenerative nature of the disease, development of a range of motor and non-motor symptoms (NMSs), and disease-related disability [2]. Henceforth, ET has gained recognition as a heterogeneous family of diseases with both motor and non-motor features. In addition to action tremor – a core motor symptom of the disease – many motor features (other types of tremor, bradykinesia, cerebellar dysfunction, balance and gait abnormalities) and NMSs (cognitive deficits, neuropsychiatric symptoms [anxiety and depression, specific personality traits], sensory deficits [hearing impairment and olfactory dysfunction] and others [sleep disorders]) have been added to disease-related symptomatology [3,4]. In tandem with the increasing knowledge of clinical symptoms, our understanding of anatomical localization and pathological basis for the tremor has improved [5]. Though the underlying neuropathology of the disease has not yet been fully elucidated, numerous clinical, neuroimaging, and histopathological researchers have continued to link ET to the dysfunction, and likely degeneration, of the cerebellum and its connections [6,7,8,9].

Hearing impairment and especially olfactory dysfunction have long been associated with neurodegenerative diseases such as Parkinson’s disease, Alzheimer’s disease, multiple sclerosis, Huntington’s disease, spinocerebellar ataxias, and motor neuron disease [10,11,12]. Taking into account this strong association, researchers have investigated the possible occurrence of hearing impairment and olfactory dysfunction in ET patients. In this article, I review and summarize (i) the evidence for their presence/absence and (ii) their potential mechanistic basis.

## Method

### Search strategy

A PubMed literature search was performed in the May 2019 database using the key words essential tremor AND sensory deficit, ‘essential tremor’ AND ‘olfaction’ OR ‘olfactory dysfunction’ OR ‘smell’ OR ‘hyposmia’ OR ‘anosmia’, ‘essential tremor’ AND ‘hearing impairment’ OR ‘hearing loss’ OR ‘hearing dysfunction’ OR ‘hearing’ OR ‘deafness’. The reference list of relevant articles was also searched to identify studies that were missed in the search process.

### Selection criteria

**Figure 1:**
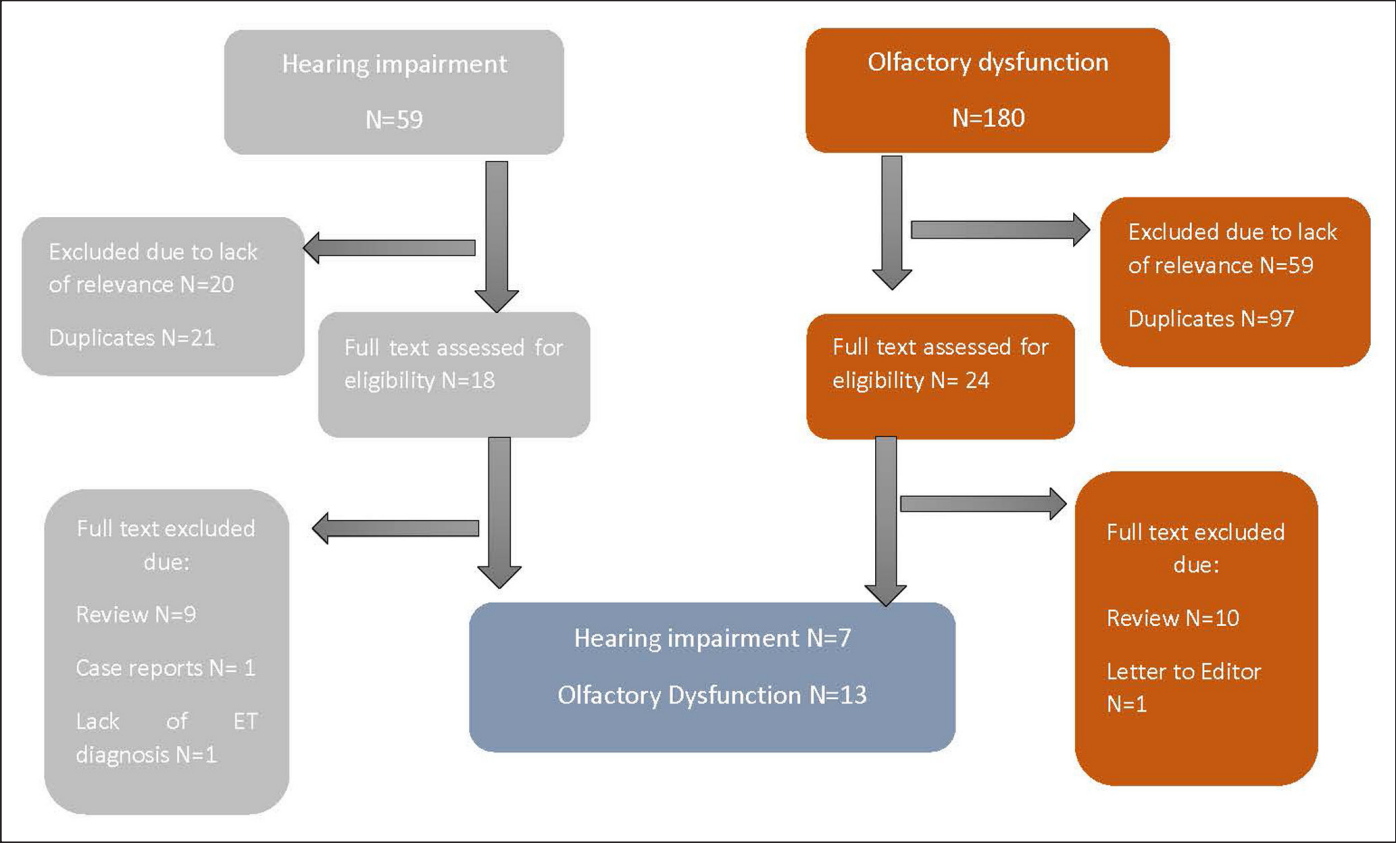
Flow Chart of the Study Selection Process*. * Sensory deficits: Five article detected: Two were excluded due to lack of relevance, 2 were review, 1 article was duplicate.

The search was limited to English language publications. All articles without case-control design such as case reports, review articles, and letters to the editor were excluded (Figure 1).

## Results

### 1. Auditory System

Hearing is an essential sense for communication, socialization, and autonomy, in addition to being a vital system for environmental awareness. Hearing impairment is associated with cognitive decline, depression, increased risk of dementia, poor balance, falls, hospitalizations, and early mortality [13].

#### 1.1. The anatomy of the auditory system

**Figure 2:**
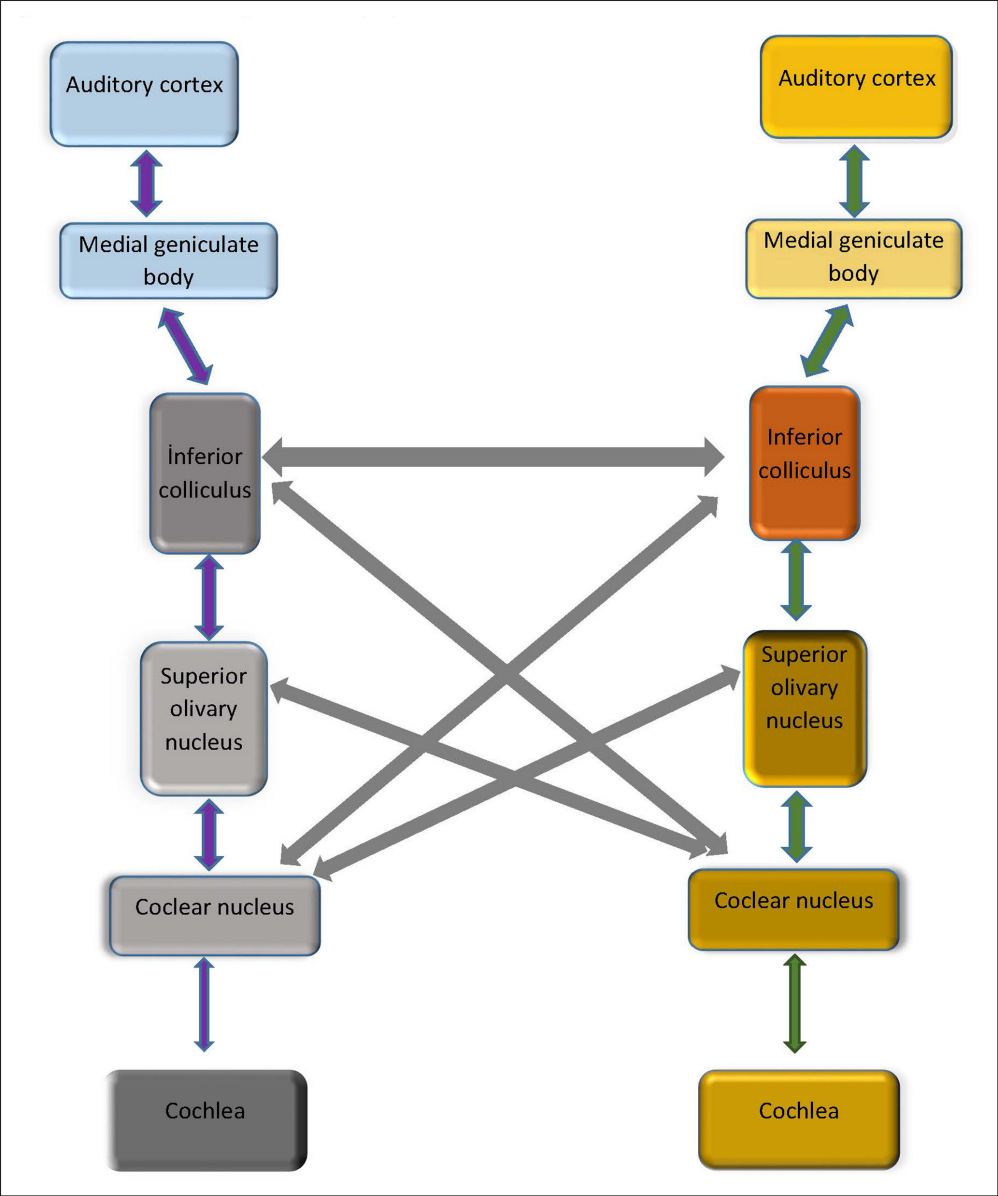
Neuroanatomy of Auditory System.

The auditory system can be divided into two systems: the peripheral system and the central system [14]. The peripheral system includes the outer ear, the middle ear, the auditory nerve and the cochlea with the organ of Corti, which contains hair cells as auditory receptors. Receptors sensitive to high frequencies are located near the cochlear base and those sensitive to low frequencies are located near the apex of the cochlea. The hair cells are innervated by the peripheral processes of bipolar ganglion cells in the spiral ganglion. Their central processes form the cochlear division of the vestibulocochlear nerve and terminate in the cochlear nuclei [15]. The peripheral auditory system is located, for the most part, in the temporal bone and the central auditory system is located in the brain. The central auditory system includes the cochlear nucleus, the superior olivary complex, the lateral lemniscus (both nuclei and pathways), the inferior colliculus, the medial geniculate body (MGB), the auditory subcortex (subcortical white matter and basal ganglia region), the cortex, and the interhemispheric pathways (including the corpus callosum). The auditory subcortex and cortex involve structures such as the internal capsule, the insula, Heschl’s gyrus, the planum temporale, and other parts of the superior temporal gyrus. Auditory responsive areas also include segments of the frontal lobe, the parietal lobe, the angular gyrus, the supramarginal gyrus, and the corpus callosum [14] (Figure 2).

#### 1.2. Evaluation of auditory system

Hearing loss categorized by the area of pathology. Conductive hearing loss is related to defects in the conductive mechanisms in the middle ear, resulting from various conditions, such as otitis media and otosclerosis. Sensorineural hearing loss is caused by diseases in the cochlea or its central connection. This type of hearing loss is divided into two subcategories, as cochlear and retrocochlear. Hearing loss that has both conductive and sensorineural components is categorized as mixed. Pathologies that originate from the VIIIth nerve, brainstem, and their projections are classified as retrocochlear [14]. Central hearing loss related with central auditory system deficits. Central auditory processing includes localization and lateralization, discrimination of speech and non-speech sounds, auditory pattern recognition, temporal aspects of audition, including integration, resolution, ordering, and masking, and auditory performance with competing and/or degraded acoustic signals [16].

Routine audiologic test batteries include pure-tone audiometry, immitancemetry and optoacoustic emissions. The cornerstone of audiologic testing is the pure-tone audiogram (PTA), which is used as a screening test for hearing loss. The aim is to establish the absence of hearing loss, hearing thresholds and, if abnormal, to distinguish between conductive and sensorineural hearing loss [17]. Middle ear function tests include “impedance audiometry” and “acoustic reflex testing,” which are designed to evaluate the sound-transmitting properties of the middle ear bones and the auditory nerve, the function of the Eustachian tube and middle ear muscles, as well as middle ear pressure. Short increment sensitivity index (SISI), transient otoacoustic emission, and tone decay are the tests that evaluate cochlear pathology. Otoacustic emissions (OAE) are the tiny sounds that arise from outer hair cells. Two types of OAE measurements are used in audiology clinics to evaluate cochlear status: Transient evoked otoacoustic emissions (TEOAE) and distortion-product otoacoustic emissions (DPOAE) [18]. Speech audiometric testing includes speech discrimination, recognition, threshold, maximum performance for single-syllable words, sentence materials with ipsilateral competition, and a dichotic sentence task [19]. Auditory evoked potentials are electrophysiological responses that are used to assess auditory function and neurological integrity. Auditory evoked responses can be subdivided according to their latency into the brain stem (ABR), middle latency (MLR) and cortical (ACR) responses [20].

#### 1.3. Hearing impairment and neurodegenerative diseases

A large number of studies have demonstrated an association between hearing loss and neurodegenerative diseases [21,22,23,24,25,26,27,28,29,30,31,32,33]. Hearing loss is a risk marker for cognitive decline and dementia [23]. Both peripheral and central auditory system dysfunction occur in the prodromal stages of Alzheimer’s Disease (AD) [24]. The reverse causality might be possible between dementia and hearing impairment [25,26]. Previous studies also reported greater hearing loss and impaired auditory processing in PD patients [27,28]. However, according to another approach, similar patterns of sensorineural hearing loss (slightly worse in the PD group) were observed when comparing the control group and PD patients, which were typical for their age [29]. Auditory dysfunction is also shown in Huntington’s disease, diseases linked to the cerebellum, such as spinocerebellar ataxias, and Friedreich’s ataxia [30,31,32,33].

#### 1.4. Hearing and cerebellum

The cerebellum has many connections, including those to the auditory pathway. The inferior colliculus has connections with the cerebellum via the dorsolateral pontine nucleus (DLPN). The MGB of the thalamus and the auditory cortex have also been shown to communicate indirectly with the vermis of the cerebellum via the DLPN [34,35].

Some forms of cerebellar ataxia may present with abnormalities in the audiological evaluation, again including the pathology of both peripheral and central auditory pathways. The most evident abnormalities in the audiological evaluation include impedance audiometry, absence of acoustic reflex, and an increase in the latency or absence of waves I, II and V and of the interpeak intervals I–III, I–V, and III–V in electrophysiological evaluation [36,37].

#### 1.5. Hearing impairment and essential tremor

Sensorineural hearing loss and hearing aid usage are common among ET patients. A limited number of studies have tried to elucidate this association (Table 1).

**Table 1:** Hearing Impairment Studies Characteristics and Design.

*Study, year*	Method of the study/Type of Test	Number of Subjects	Mean Age (y)	Gender M/F	Duration of Tremor (y)	Test Results	P value

***Ondo et al. 2003* [38]**	NHHI -Describing hearing loss (DHL) -Hearing aid usage (HAU) Acoustic immittance measures (tympanogram, crossed and uncrossed acoustic reflexes) Distortion-product otoacoustic emissions Pure tone air-conduction Audiometry Speech audiometry (speech threshold, PBm, SSIm, DSI) Bone-conduction audiometry	250 ET, 127 PD, 127 HC 74 ET (10 patients excluded because of middle ear disease that was identified during audiometry testing)	66.2 ± 13.5 65.3 ± 9.6 62.6 ± 11.9	138/112 68/59 66/61	24.8 ± 17.9 7.9 ± 5.6 NA	Total NHHI scores differed between groups DHL: Scores were highest in the ET group followed by the control group and patients with PD HAU: 16.8% in ET 1.6% in PD 0.8% in HC Higher frequency loss was detected in ET group.	ET vs. HC: <0.001 ET vs PD: <0.001 <0.0001
***Louis et al. 2005* [39]**	Brain repository study Reported deafness	94	74.1 ± 10.1	38/57	40 ± 20.3	20 (21.3%) of 94 ET cases, including 18 (26.9%) among the 67 participants ≥70.18 patients were using hearing aid.	
***Benito-Leon et al. 2007* [40]**	Population based study, subjects with >65 years’ old Reported hearing impairment	248 ET 4669 Controls	75 73	104/144 1985/2684	5	38.7% vs 29.4% After adjustments for age, gender, educational level, and depressive symptoms, there was an association between hearing impairment and ET	0.002 0.021 OR =1.4 95% CI = 1.05–1.8
***Balaban et al. 2011* [42]**	Pure tone audiometric test (PTA) Tympanogram Transient-evoked otoacoustic Emissions (TEOAE) Auditory brainstem response (ABR) Bitermal caloric test.	23 ET 21 HC	49.4 ± 26.4 51.9 ± 24.1.	17/6 11/10	7.86 ± 6.39 NA	PTA: 30 ± 23.3 vs. 22.5 ± 15.2 The average hearing thresholds at frequencies of 250 and 500 Hz were found to be higher in ET. The TEOAE responses of the ET patients and HC were found significantly different. ABR (I,V peak, I-V interpeak latencies did not differed between groups	<0.05 <0.05 <0.05 >0.05
***Benito- Leon et al. 2011* [41]**	Reported hearing impairment	207 ET 2472 Controls	76.0 75.3	99/118 968/1404		44.9 vs. 36.3	<0.05
***Yilmaz et al. 2015* [43]**	Described hearing loss by subjects PTA Speech recognition threshold, Tympanogram, Short increment sensitivity index (SISI), Tone decay, TEOAE	34 ET 45 HC	57.5 60.0	16/18 20/25	12 NA	50% vs. 2.2 The tone decay mean values in 4,000 Hz of the patients’ right ear were significantly higher. The number of patients without OAE was significantly higher than in HC	<0.001 >0.05 >0.05 >0.05 <0.005 <0.05
***Ghika et al. 2015* [44]**	Assessments was made by using a questionnaire (four questions)	121 ET 54 ET-PD	66.9 72.6	52/69 24/30	13.7 18.4	65.3% vs. 28.3%	<0.001

NHHI: Nursing Home Hearing Handicap Index. PBm: Maximum performance for single syllable words SSIm: Synthetic Sentence Index DSI: Dichotic Sentence Index.

The first hearing impairment investigation in ET evaluated 250 patients with ET, 127 patients with PD, and 127 healthy controls (HCs) by using the Nursing Home Hearing Handicap Index (NHHI), a 10-question survey designed to elicit evidence of hearing impairment. A subgroup of 64 ET patients was recruited for complete audiometric evaluation, including acoustic immittance measures (tympanogram, crossed and uncrossed acoustic reflexes), distortion-product otoacoustic emissions, pure tone air-conduction audiometry, and speech audiometry. The percentage of hearing aid usage was 16.8% in ET, 1.6% in PD, and 0.8% in HCs. After adjustments for sex and age, NHHI scores were higher in the ET group than in both the PD and HC groups (p < 0.001, p < 0.001). The audiometric evaluation showed high-frequency sensorineural hearing loss in ET patients. Considering the neuropathology of ET, they mentioned two possible underlying pathologies: 1. Hearing abnormalities might be explained by abnormalities in cerebello-thalamo-cortical pathways or impaired integrity of the auditory pathway including the MGB located in the ventral thalamus. 2. Mutation of the connexin protein which has been previously associated with tremor [38].

In a brain repository study, *Louis et al*. found that a complaint of “deafness” occurred in 20 (21.3%) of 94 ET cases, including 18 (26.9%) among the 67 participants aged 70 or older (All 18 wore hearing aids) [39]. But there was no control group due to the nature of this study. Two large population-based studies from Spain done by the same group (40,41) revealed an association between reported hearing impairment and ET. Of the patients aged >65 years, 38.7 % of patients reported hearing impairment compared to 29.4% of controls (p = 0.002), after adjustments for age, sex, educational level, depressive symptoms, and dementia. The study showed that participants who reported hearing impairment were 30% more likely to suffer from ET than the controls (OR 1.3; 95% CI 1.01–1.7; p = 0.04) [40]. Taken together, these population-based studies provide support for the association between hearing impairment and ET.

In the study of *Balaban et al.*, the audio-vestibular system in ET patients was evaluated using the following audiologic tests: pure tone audiometric test, tympanogram, TEOAE, ABR, and bithermal caloric test for the evaluation of the vestibular system. In this study, patients with ET exhibited a significant elevation of PTA threshold in 250 and 500 Hz frequencies, and abnormal TEAOE results with no correlation between tremor severity or tremor duration and audiometric scores. ABR was not different between groups. They concluded that the abnormalities are due to the cochlea rather than an auditory brainstem pathway pathology, which is responsible for hearing loss associated with ET [42]. *Yilmaz et al*. evaluated sensorineural hearing loss by PTA, speech recognition threshold, tympanogram, SISI, tone decay, and otoacoustic emission audiological tests in non-depressed subjects. 50% of the ET group and 2.2% of HCs described hearing loss (p < 0.001). The tone decay test values were found to be significantly higher for the right ears of patients at 4,000 Hz compared to the control group. The percentage of the patients in whom OAE could not be obtained in the right ear was significantly higher than in the control group (38.2% and 32.3%, p < 0.05) but this was not the case in the left ear (13.3% and 15.6%, p = 0.14). Although different results in both ears remain a question mark, the researchers agreed with the previous study’s results about cochlear hearing loss. They associated cochlear pathology with hairy cell loss, which might be a result of connexin mutations [43].

One study compared hearing impairment between ET, and patients with ET PD based on questionnaires. Results of this study were quite interesting. The prevalence of patients who describe hearing impairment was 65.3% in ET vs. 28.3% in patients with ET PD (p < 0.001) [44]. This results indicates that hearing impairment is more specific to ET.

#### 1.6. Hearing impairment: conclusions and implications

The relationship between hearing impairment and ET is gaining acceptance, but the underlying pathology is still unclear. Two possible explanations have been suggested: 1. Peripheral pathologies, and 2. Central pathologies.

Recent data supports high frequency hearing loss which might be a result of a cochlear pathology. The relationship between cochlear hearing loss and ET could be associated with connexin mutations. Connexin proteins are involved in a number of pathological conditions in humans, mainly in hearing loss and neurodegenerative disorders [45,46]. However, it is shown that connexin mutations are also related to retrocochlear auditory centers [47]. Other than one research which evaluated the brain stem via ABR, there is no study investigating the central parts of the auditory system. Considering the neuropathology of the cerebellum and its connections to the inferior colliculus and ventral thalamus (MGB), it is conceivable that an impaired central auditory pathway is a cause of hearing dysfunction in ET. Investigation of the integrity of the auditory pathway from brainstem to cortex is needed to increase our knowledge on the basis of hearing impairment in ET.

In conclusion, the data we have on the subject is mostly based on studies that have evaluated the prevalence of hearing impairment in ET. Two studies revealed cochlear pathology. One of them evaluated the integrity of the brainstem and found no pathology. Presence of retrocochlear and central auditory processing pathologies have not yet been extensively evaluated. Contribution of central auditory system pathologies in hearing impairment needs to be defined.

### 2. Olfactory System

The olfactory sensory system is a remarkable circuit of the brain. Contrary to our other senses, it is the only one that directly reaches the cortex without thalamic relay (48). In addition to having a vital function, such as a warning of dangerous situations (avoiding spoiled food, detecting smoke) even during sleep, the sense of smell also plays a vital role in emotion, behavior, memory, and personal interactions [49,50].

#### 2.1. The anatomy of the olfactory system

**Figure 3:**
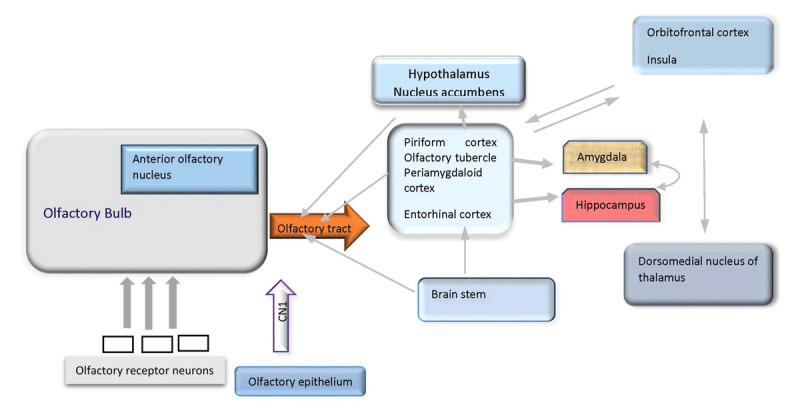
Neuroanatomy of Olfactory System.

Primary olfactory neurons are located in the olfactory epithelium of the upper nasal cavity. Their axons form the fila olfactoria, which cross the cribriform plate entering into the anterior cranial fossa, ramify in the most superficial layer of the olfactory bulb. The olfactory bulb continues to the olfactory tract and arrives at the primary olfactory cortex areas, such as piriform cortex (which has connections to insular cortex through the thalamus, orbitofrontal cortex, hypothalamus), amygdala, anterior olfactory nucleus, and entorhinal cortex (cortical input is relayed to hippocampus through the entorhinal cortex) [51,52] (Figure 3).

#### 2.2. Evaluation of the olfactory system

Evaluation of the olfactory system consists of three main domains: 1. The olfactory threshold, which is a measure of the lowest concentration of an odorant that activates the olfactory receptor cells, 2. Odor identification, which is the ability to identify odorants, and 3. Odor discrimination, which is the ability to differentiate between odorants. The olfactory threshold is mostly associated with peripheral parts, while others are mostly provided by the central parts of the olfactory system. The most popular and the most suitable identification tool for research is the University of Pennsylvania Smell Identification Test (UPSIT-40) comprising 40 different odors. Cultural factors, however, could affect odor identification [53]. The UPSIT includes components which non-Americans may not be familiar with. Using the smaller International UPSIT-12 kit can be the solution. The Connecticut Chemosensory Clinical Research Center Test (CCCRC Test) and the Sniffin’ Sticks Test (SST) are other reliable tests [12].

Olfactory event-related potentials (OERPs) is an electrophysiological technique which allows the evaluation of changes in numerous brain areas, starting from amygdala and regions of the medial temporal lobe, followed by the mid-orbitofrontal cortex and insular cortex, along with regions of the temporal lobe. Another useful technique is functional magnetic resonance imaging in order to study the functional neuroanatomy of the olfactory system [12].

When interpreting research that is focused on the olfactory system in humans, it is crucial to keep in mind that 1. Normal aging is also found to be related to olfactory dysfunction [54], and 2. There is a sex-related difference favoring women [55].

#### 2.3. Olfactory dysfunction and neurodegenerative diseases

A deterioration in olfactory function has become one of the most prevalent deficits among different neurodegenerative disorders. Olfactory dysfunction is a common and early feature of AD and Lewy body diseases, PD, and Parkinson’s disease dementia [56,57].

In AD, olfactory dysfunction is present in up to 90 % of patients. All three components of olfaction (odor identification, discrimination, and threshold) are impaired, and neuropathological changes have been shown in the peripheral and central olfactory structures [58]. The degenerative process in AD is characterized by plaques and neurofibrillary tangles that start in the entorhinal cortex and then proceed to other temporal lobe structures, including the hippocampus (according to Braak’s staging) [59]. One of the first damaged areas in AD brains is the transentorhinal cortex, which is a structure involved in olfaction. As a consequence, olfactory dysfunction is an early sign of AD and can predict the conversion of mild cognitive impairment (MCI) to AD [60,61,62].

Olfactory dysfunction is also one of the main symptoms of PD [63]. Odor detection is damaged in about 75% of PD patients, while odor identification is impaired in 90% [64]. Olfactory impairment appears years before motor symptoms occur [65,66,67]. This confirms that the earliest pathological changes in stage I (Braak staging) occur in the olfactory bulb and the anterior olfactory nucleus, while the substantia nigra is not involved until stage III [58].

Additionally, other neurodegenerative diseases such as frontotemporal dementias (FTD), corticobasal degeneration (CBD), progressive supranuclear palsy (PSP), multiple system atrophy (MSA), amyotrophic lateral sclerosis (ALS), multiple sclerosis and Huntington’s disease (HD), X-Linked Dystonia–Parkinsonism, Down syndrome, and schizophrenia also show varying degrees of olfactory dysfunction [54].

#### 2.4. Cerebellum and Olfaction

The cerebellum is classically viewed as a primary motor control organ with specific roles in coordination and motor learning, and is structurally connected with all major subdivisions of the central nervous system, including the cerebrum, basal ganglia, diencephalon, limbic system, brainstem, and spinal cord [68]. Insights from both lesion and neuroimaging studies verify the view that the human cerebellum not only has functions in motor control, but also supports various cognitive, emotional, and behavioral functions, as well as olfaction [69]. Animal and functional MRI studies, reports of olfactory dysfunction in patients with cerebellar lesions (focal cerebellar stroke, tumor resections), association of cerebellar disorders (degenerative ataxias, multisystem atrophy, schizophrenia, etc.) and olfactory dysfunction indicate that the cerebellum has a role in the olfactory system [70,71,72].

Many researchers have presented that there is a mild but significant impairment of olfaction in hereditary ataxias, in which the main clinical manifestations result from the involvement of the cerebellum and its connections, such as Friedreich ataxia and spinocerebellar ataxia type 2, 3, 7 and 10 [68,73,74,75,76,77,78].

#### 2.5. Olfactory dysfunction and essential tremor

As with many other neurodegenerative diseases, olfactory functions have been investigated in ET [79,80,81,82,83,84,85,86,87,88,89,90] (Table 2).

**Table 2:** Olfactory Identification Studies Characteristics and Design.

*Study, year*	Type of Test	Number of Subjects	Mean Age (y)	Gender M/F	Duration of Tremor (y)	Test Results	P value			

***Busenbark et al, 1992* [79]**	UPSIT	16 ET				36.3 vs.37.2	0.19
		17 HC					
***Louis et al, 2002* [80]**	UPSIT	37 ET	68.9	16/21	18.9	29.0 ± 6.1 vs	0.02
		37 HC	67.3	18/19	NA	31.9 ± 4.6	
***Louis et al, 2003* [82]**	UPSIT	13 ET with RT	73.7	7/6	24.9	29.3 ± 4.3 vs	0.69
		58 ET no RT	66.6	28/30	22.0	29.4 ± 6.4	
***Applegate et al, 2005* [81]**	UPSIT	87 ET	67.8	39/46	22.5	29.2 ± 6.6 vs.	0.04
		92 HC	67.1	41/51	NA	31.3 ± 5.4	
***Shah et al, 2008* [84]**	UPSIT	245 HC	49.5	84/161	NA	33.0	
		64 Tremulous PD	67.2	44/20	4.8	18.05	<0.001*
		30 Familial ET	53.1	15/15	24.2	35.0	<0.001*
		29 Non familial ET	67.3	12/17	12.4	31.0	0.67*
***Louis et al, 2008* [83]**	UPSIT	Total 83 ET	63.7	55/38	20.7	30.5	0.09**
		40 ET with HBHC					0.002***
		43ET with LBHC					
		69 HC	63.5	31/38	NA	31.7	
***Djaldetti et al, 2008* [86]**	UPSIT	7 ET	53		8.5	23.2	NSv
		17 Mixed tremor	72		15.4	21.7	NS*
		17 PD	61		6.7	13.7	<0.001*
		9 HC	53		NA	27.2	
***Silveira-Moriyama et al, 2009* [87]**	UPSIT	21 SWEDD	65.4	10/11	9.3	27.3	0.07*
		26 ET	69.0	8/18	11.4	27.9	0.4*
		16 dystonia	66.7	7/9	19.7	27.6	0.9*
		191 PD	65.6	114/77	10.2	17.6	<0.001*
		136 HC	64.9	72/64	NA	29.5	
***Quagliato et al. 2009* [85]**	UPSIT-12	40 ET(21 definite, 11 possible, 8 probable ET)	59.8	17/23	17.4	9.10 vs. 9.11	Undetermined
		89 HC	56.08	34/55	NA		
***McKinnon et al. 2010* [88]**	UPSIT	207 HC	77.0	59/158	UD	29.7	<0.001**** >0.05*
		23 Suspected PD	80.8	11/12		28.1	>0.05**
		15 Possible PD	81.7	7/8		27.0	>0.05
		19 Probable PD	71.7	12/7		20.7	<0.001*
		37 ET	79.4	20/17		31.2	>0.05*
		25 RLS	74.5	6/19		32.7	>0.05*
		27 MCI	81.3	19/14		26.6	>0.05*
***Bradvica et al. 2015* [90]**	Pocket Smell Test PST						<0.001****
		51 ET	65.2	17/34		19.6%	
		59 PD	67.2	35/24		74.6%	
		26 HC	60.2	13/13		23.1%	
***Wu et al. 2016* [89]**	Sniffin Sticks (SS-16)	49 ET	UD	UD	UD	9.47	0.82
		79 HC				9.66	

* Comparing HC. ** Comparison of transformed UPSIT score. *** Comparison of HBHC and LBHC groups. **** For null hypothesis. ET: Essential tremor, HC: Healthy controls, NA: Not applicable, RT: Resting tremor, PD: Parkinson’s Disease, HBHC: High blood harmane concentration, LBHC: Low blood harmane concentration, NS: Not significant, RLS: Restless legs syndrome, MCI: Mild cognitive impairment.

In the first cross-sectional study on the subject of olfactory function in ET, the researchers compared UPSIT scores between ET, tremor dominant PD and HCs. Tremor dominant PD patients had significantly worse performance on UPSIT but ET cases did not, suggesting that olfaction may be useful to distinguish ET from tremor dominant PD [79]. After growing evidence that ET is a neurodegenerative disorder**, studies in this area have gained momentum. *Louis and colleague*s conducted a study with a relatively larger sample size (37 ET cases and 37 HCs) that revealed lower olfaction scores in ET patients compared to HCs (29.0 ± 6.1 vs. 31.9 ± 4.6, *p* = 0.02). The UPSIT score was not correlated with tremor severity [80]. The same group of authors further extended their study to 87 ET patients to consider the effects of mild cognitive deficits and reported similar low UPSIT scores that also were not correlated with tremor severity [81]. These two studies suggested mild but significant olfactory dysfunction in ET patients.

Comparing ET patients with and without rest tremor, there were no differences between the groups (29.3 ± 4.3 vs. 29.4 ± 6.4; p = 0.69). In addition to this, the scores were higher than 95% of PD patients. This result identified that ET patients with rest tremor may not have early PD but it should be considered a form of ET [82].

Harmane is a toxic chemical for the cerebellum which might lead to ET. A study revealed that higher log blood harmane concentration was correlated with lower UPSIT score (rho = –0.46, p < 0.001). This study was instrumental in highlighting the role of the cerebellum in the pathology of ET and olfaction [83].

*Shah and colleagues* compared olfactory functions by UPSIT and olfactory event-related potentials (OERPs) between three groups: 59 ET patients, 64 tremor dominant PD patients, and HCs (245 HC for the comparison of UPSIT, 74 HC for the comparison of OERPs). The results showed that olfactory testing by UPSIT or OERP was normal in ET, although the scores of non-familial ET were lower than HCs, it was not statistically significant and there was a marked difference between ET (whether familial or not) and tremor-dominant PD. Patients with PD scored worse compared to controls and against both the familial and non-familial ET groups [84]. Similar findings were reported in a Brazilian study. *Quagliato and colleagues* compared 40 ET patients (21 of the cases had definite ET diagnosis, the remaining were possible or probable ET cases) with 89 HCs using UPSIT-12. The mean UPSIT-12 score was 9.0 in the ET group, while it was 9.1 in the HC group. The study did not compare definite ET cases to HCs, but compared groups according to their age range. The same research group also conducted this study with PD patients. In the PD group, the mean UPSIT score was 5.7. They concluded the normality of olfactory identification in ET, qualifying UPSIT to be an important tool for the differential diagnosis of a tremor with undetermined origin [85].

*Djaldetti et al*. studied olfactory function in patients with combined rest and postural tremor. The results showed that most of these patients had normal SPECT imaging. They showed that ET patients had olfaction scores similar to those of HCs and the mixed tremor group, but higher than the PD group [86]. *Silveira-Moriyama et al*. evaluated olfaction in patients without evidence of dopaminergic deficit (SWEDDS) in their scans. The study showed that the scores of patients with ET were comparable to the SWEDDS, dystonia, and HC [87]. Results of both studies indicated that patients with PD had lower scores than the other tremor groups [86,87].

*Mckinnon and colleagues* compared olfaction in a group of subjects consisting of PD, ET, restless legs syndrome (RLS), mild cognitive impairment (MCI), and HCs, and again tested the hypothesis that olfactory dysfunction might differentiate PD from others. The clinically probable PD group was the only group to have hyposmia. However, there were no differences between the other groups (Clinically suspect PD, clinically possible PD, ET, RLS, or MCI). Interestingly, this result did not support previous literature about olfactory deficits occurring in early MCI. Similarly, two studies published in recent years found the occurrence of normal olfactory function in ET [89], which might be helpful in differential diagnoses [90].

#### 2.6. Olfaction: conclusions and implications

Although some studies demonstrated mild olfactory dysfunction, the current literature does not support olfactory dysfunction as a primary feature in patients with ET. This could be related to the heterogeneity of the disease. One study found a relationship between high blood harmane concentration and olfactory dysfunction. This result shows the importance of environmental factors on olfactory function in ET. Most previous studies did not compare ET patients according to family history, age of onset, or ET and ET-plus. New research considering the disease heterogeneity is needed to clarify the association. Currently, the data supports olfactory dysfunction can be useful for distinguishing between ET and PD.

## Conclusion

ET is a heterogeneous neurodegenerative family of diseases with a broad spectrum of motor and non-motor features. As in other neurodegenerative diseases, researchers have investigated the relationship between ET and sensory deficits such as hearing impairment and olfactory dysfunction. Through a limited number of studies, the research has shown an association with hearing impairment and tried to define the underlying cause. More studies that include central parts of the auditory system are needed. Reports on olfactory dysfunction have conflicting results. Considering the role of the cerebellum on olfaction and the association between cerebellar neurodegenerative disease and olfactory dysfunction, the presence of mild olfactory dysfunction in ET is possible. Conflicting results may be due to the lack of consideration of the disease’s heterogeneity. Comparing subgroups of patients may be a useful method to combat this limiting factor. Further work is needed in order to understand the prevalence and underlying reasons of these sensory features.
